# A Comparison of Techniques for ^90^Y PET/CT Image-Based Dosimetry Following Radioembolization with Resin Microspheres

**DOI:** 10.3389/fonc.2014.00121

**Published:** 2014-05-22

**Authors:** Alexander S. Pasciak, Austin C. Bourgeois, Yong C. Bradley

**Affiliations:** ^1^Department of Radiology, The University of Tennessee Medical Center, Knoxville, TN, USA; ^2^Department of Radiology, The University of Tennessee Graduate School of Medicine, Knoxville, TN, USA

**Keywords:** yttrium-90 radioembolization, image-based dosimetry, yttrium-90 PET/CT, yttrium-90 dosimetry, radioembolization dosimetry

## Abstract

^90^Y PET/CT following radioembolization has recently been established as a viable diagnostic tool, capable of producing images that are both quantitative and have superior image quality than alternative ^90^Y imaging modalities. Because radioembolization is assumed to be a permanent implant, it is possible to convert quantitative ^90^Y PET image sets into data representative of spatial committed absorbed-dose. Multiple authors have performed this transformation using dose-point kernel (DPK) convolution to account for the transport of the high-energy ^90^Y β-particles. This article explores a technique called the Local Deposition Method (LDM), an alternative to DPK convolution for ^90^Y image-based dosimetry. The LDM assumes that the kinetic energy from each ^90^Y β-particle is deposited locally, within the voxel where the decay occurred. Using the combined analysis of phantoms scanned using ^90^Y PET/CT and ideal mathematical phantoms, an accuracy comparison of DPK convolution and the LDM has been performed. Based on the presented analysis, DPK convolution provides no detectible accuracy benefit over the LDM for ^90^Y PET-based dosimetry. For PET systems with ^90^Y resolution poorer than 3.25 mm at full-width and half-max using a small voxel size, the LDM may produce a dosimetric solution that is more accurate than DPK convolution under ideal conditions; however, image noise can obscure some of the perceived benefit. As voxel size increases and resolution decreases, differences between the LDM and DPK convolution are reduced. The LDM method of post-radioembolization dosimetry has the advantage of not requiring additional post-processing. The provided conversion factors can be used to determine committed absorbed-dose using conventional PET image analysis tools. The LDM is a recommended option for routine post-radioembolization ^90^Y dosimetry based on PET/CT imaging.

## Introduction

The complexity of techniques for ^90^Y radioembolization treatment-planning and post-infusion dosimetry has grown significantly in recent years. Methods of independently characterizing dose to both the target tumor and non-target hepatic parenchyma first emerged with compartmental dosimetry models (1) and the Medical Internal Radiation Dose (MIRD) schema for ^90^Y hepatic radioembolization (2). From basic two-compartment models, research in ^90^Y radioembolization has evolved toward 3-dimensional (3-D) absorbed-dose maps, which can be utilized for analysis of dose-volume histograms (DVH) and comparison of tumor-absorbed-dose with known tumoricidal thresholds.

^90^Y PET/CT has recently been shown to be an alternative to traditional post-infusion ^90^Y imaging methods. ^90^Y PET not only provides superior image quality to alternative modalities, but imaging is also quantifiable to a high degree of accuracy (3). Image-based dosimetry for radioembolization has been performed and results reported in the literature both with and without the use of ^90^Y PET/CT using dose-point-kernel (DPK) convolution. Kennedy (4) and Dieudonne (5) have used 3-D SPECT data following infusion of ^99m^Tc MAA translated into absorbed-dose maps using DPK convolution. Numerous authors have used DPK convolution as a tool to compute absorbed-dose in both patient and phantom studies using post-infusion imaging with ^90^Y PET/CT (6–11). In several cases, dosimetry was performed using DPKs from published literature such as reported by Strigari (12) or Lanconelli (13). However, because the DPK voxel size must match the PET acquisition matrix voxel size, DPKs were often calculated for specific scenarios using Monte Carlo radiation transport codes. Many Monte Carlo codes have been employed in the ^90^Y PET literature for this purpose including EGSnrc (4), MCNP-X (10), and FLUKA (9). These techniques are further detailed in a review of ^90^Y PET/CT imaging and applications provided by Pasciak et al. (3).

In a previous study (14), it was shown experimentally that dosimetry for ^90^Y radioembolization based on the hepatic distribution of ^99m^Tc MAA as obtained from SPECT imaging was most accurately performed using a technique called the Local Deposition Method (LDM) rather than DPK convolution. In the LDM, ^90^Y β-particles released by decay within a voxel deposit energy locally, within the same voxel. Using the LDM, absorbed-dose in each voxel can be determined by multiplying activity concentration by a constant scalar factor. Pasciak and Erwin (14) confirmed that because of the limited resolution of SPECT, the blurring of the true activity distribution by the point-spread-function (PSF) of the imaging system in-effect spread the dose out more than convolution with the ^90^Y DPK. As such, image-based dosimetry using DPK convolution was less accurate than the LDM, owing to the fact that the activity concentration was already blurred by the scanner. The reduced accuracy of DPK convolution was greatest as voxel size decreased and matrix size increased. LDM was adopted for clinical dosimetry based on ^99m^Tc MAA by Chiesa et al. (15) and Mazzaferro et al. (16). However, only Kao et al. (17) and Bourgeois et al. (18) have published clinical results of ^90^Y PET scans utilizing dosimetric data produced by the LDM without the use of DPK convolution.

This manuscript aims to extend the analysis of Pasciak and Erwin (14) to ^90^Y PET/CT by comparing DPK convolution with the LDM.

## Materials and Methods

Two comparisons were performed in this work to contrast DPK convolution with the LDM including: (1) an experimental comparison and (2) a mathematical comparison.

### Experimental comparison

The NEMA IEC Body PET Phantom (NEMA Phantom) was used in the experimental comparison. NEMA Phantom hot-spheres were filled with a 2.2 MBq/mL solution of ^90^Y Chloride along with 1 g/L of diethylene triamine pentaacetic acid (DTPA) to inhibit binding between the radionuclide and the phantom surface. The phantom background was filled with a ^90^Y Chloride activity concentration of 0.74 MBq/mL, with the same concentration of DTPA. Both the activity concentrations and their ratios were selected to mirror a 3:1 tumor to normal uptake ratio (T/N) commonly displayed for non-hypervascular hepatic metastasis treated with resin ^90^Y microspheres. A total of 14 phantom scans were performed on a Siemens Biograph mCT Flow (Siemens Medical Solutions USA, Inc., Knoxville, TN, USA) using two bed positions without moving the phantom so as to characterize the effect of image noise. Eight scans were performed with a 20-min scan time and six with a 40-min scan time. All scans were performed within 10 h of phantom preparation and all were corrected for decay. Multiple reconstructions using 1 iteration, 21 subsets, and time-of-flight (TOF) were performed based on previous reports suggesting that these parameters will produce the best quantification of ^90^Y PET/CT on a Siemens Biograph mCT (19, 20). The voxel size and use of PSF resolution recovery (RR) was varied for each reconstruction as detailed in Table 1. No reconstruction filter was applied and the slice width was selected to match the pixel size, creating isotropic voxels.

**Table 1:** **Reconstruction parameters for ^90^Y phantom scans**.

Experimental reconstruction number	Phantom scan duration (min)	Number of repeated scans	Voxel size (matrix size)	Reconstruction parameters
1	20	8	4^3^ mm (200 × 200)	1i21s TOF
2	40	6	2^3^ mm (400 × 400)	1i21s TOF
3	40	6	2^3^ mm (400 × 400)	1i21s TOF + RR

**Figure 1:**
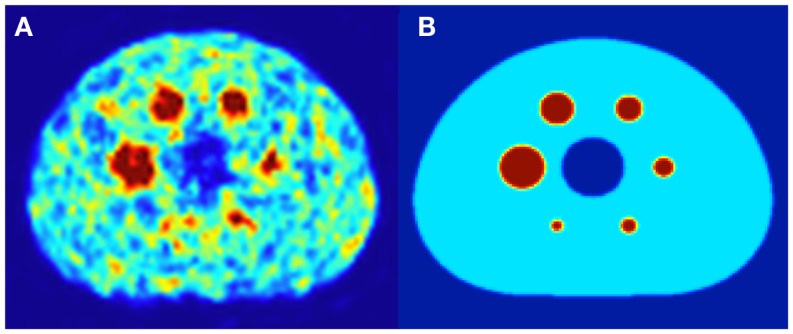
**(A)** A 4.0 mm slice through the NEMA IEC body phantom following a 20 min ^90^Y PET acquisition. The hot-sphere activity concentration was 2.2 MBq/mL, and a 3:1 ratio of activity concentration in the hot-sphere and phantom background was used to mirror typical T/N for metastatic liver cancer. **(B)** A slice through the same location in the mathematical phantom, with an identical hot-sphere to background activity concentration ratio.

Both DPK convolution and the LDM were used to perform dosimetry on all reconstructed phantom image sets (Table 1) as described in a later section. However, to analyze the validity of the two dosimetric techniques, a True Dosimetric Solution (TDS), i.e., our gold standard, was generated for comparison. A 3-D mathematical phantom was designed to match geometric specifications of the NEMA Phantom. The mathematical phantom has the benefit of being geometrically precise with no image noise. Figure 1B shows this mathematical phantom, while Figure 1A demonstrates the same slice of the NEMA Phantom from acquisition 1 in Table 1. The TDS was obtained by convolving the mathematical phantom with the DPK. The β-DPK for ^90^Y was computed using MCNP-X 2.50 (21) with isotropic voxels either 2.0 or 4.0 mm in size, matching both the mathematical and physical phantom images. Monte Carlo simulation was performed in a homogeneous medium of liver tissue as defined in a report by the International Commission on Radiation Units and Measurements (22). All convolution in this study were performed using Matlab R2013a (MathWorks, Inc., Natick, MA, USA) in three dimensions.

Modern PET/CT scanners use detectors composed primarily of cerium-doped lutetium yttrium orthosilicate (LYSO) or cerium-doped lutetium orthosilicate (LSO). Both of these materials contain lutetium, which based on natural isotopic abundance is 2.59% ^176^Lu (6). This inherent radioactivity within the LYSO/LSO PET detector blocks leads to an intrinsic low count-rate of randoms that may affect quantification accuracy at lower activity concentrations of ^90^Y (9, 19, 20). The non-negativity constraint inherent in OSEM image reconstruction also contributes to decreases in quantitative accuracy in areas of low activity concentration. High image noise can contribute to a positive bias in ^90^Y PET quantification, just as it does in dynamic PET where short acquisition frames result in high noise (3, 23). For these reasons, it is possible that the scanned activity concentration in the phantom background may experience an offset causing deviation from the nominal 3:1 activity concentration used to fill the hot-spheres and phantom background, respectively. Therefore, for each phantom scan in Table 1, the average of multiple regions of interest drawn in the phantom background were used to scale the background activity concentration in the mathematical phantom to match each physical acquisition. No scaling was done for the mathematical phantom hot-spheres since multiple authors have shown excellent quantification at the activity concentration used to fill the spheres (19, 20). The goal of this effort was to eliminate or reduce quantification inaccuracy as a measure of error when comparing the TDS and result of either DPK convolution or the LDM when applied to the scanned phantom image sets.

### Mathematical comparison

While efforts were made to eliminate quantification accuracy as a measure of error for the phantom comparison described above, image noise could not be eliminated. Although phantom scan comparisons were performed at several matrix sizes and variable scan resolution by performing reconstructions with and without point-spread function RR, it is also desirable to explore the relationship between the PET/CT scanner resolution and the relative accuracy of the LDM and DPK convolution in the absence of noise. To accomplish this, mathematical phantoms with 2.0 and 4.0 mm isotropic voxels were convolved with a range of 3-D Gaussian blur kernels to simulate the shift-invariant PSF of different PET/CT systems. Gaussian kernels with FWHM ranging from 2.0 to 15.0 mm in increments of 0.25 mm were applied to create simulated PET data. Then, either DPK convolution or the LDM was applied to the simulated PET data for subsequent comparison with the TDS.

**Figure 2:**
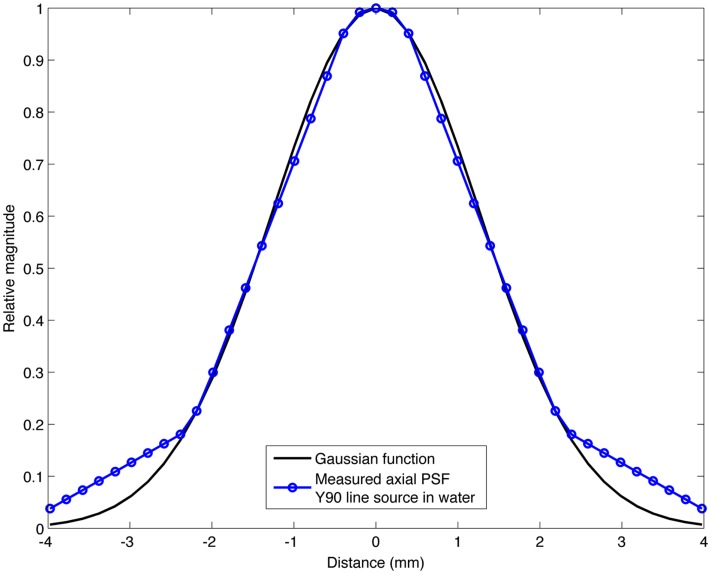
**Transaxial profile of a ^90^Y line source in water compared to a Gaussian profile with the same FWHM (3.1 mm)**.

The range of Gaussian kernels applied to create the simulated PET data exceeds the range of published FWHM of ^90^Y PET/CT data summarized in Table 2. While a Gaussian function may not exactly represent the shape of the PSF for ^90^Y PET, it is a reasonably accurate approximation. Figure 2 shows a transaxial profile of a 0.4 mm inner-diameter line source charged with Y^90^ in a cylindrical water scatter phantom placed at isocenter. Imaging was performed on the same PET/CT system used for the phantom acquisitions discussed above with a 10 cm FOV (zoom of 8), a 512 × 512 PET reconstruction matrix and 1 iteration and 21 subsets with RR and TOF. A Gaussian kernel with the same FWHM (3.1 mm) has been plotted in Figure 2 so the similarities in shape can be seen.

**Table 2:** **Published ^90^Y pet phantom imaging summary**.

Reference	Scanner	Detector material	Reconstruction parameters	Resolution (mm)
Werner et al. (24)	Siemens Biograph Hi-Rez 16	LSO	8i16s	6.4
van Elmbt et al. (25)	Phillips Gemini TF	LYSO	3i8s, TOF	9.3
van Elmbt et al. (25)	Phillips Gemini Power 16	GSO	3i8s, 8 mm Gaussian filter	10.0
van Elmbt et al. (25)	Siemens Ecat Exact HR+	BGO	3i8s, 8 mm Gaussian filter	10.6
Bagni et al. (26)	GE Discovery ST	BGO	2i15s, RR	6.5
D’Arienzo et al. (7)	GE Discovery ST	BGO	3i	5.0
Kao et al. (17)	GE Discovery 690	LYSO	3i18s, TOF, RR	10.0

### Transformation of images using DPK convolution

Unlike other forms of targeted internal radionuclide therapy, radioembolization has the benefit of negligible biological elimination following infusion. On that premise, spatial committed absorbed-dose following ^90^Y radioembolization can be calculated by 3-D convolution of the 3-D activity concentration matrix with the β-DPK for ^90^Y. Equation 1 below describes this process:

(1) $$\begin{array}{llll} D\left(x,y,z\right) & =\frac{1}{\lambda }\left(A⊗\text{DPK}\right)\left(x,y,z\right)\; & & \;\\ & =\frac{1}{\lambda }\sum_{x^{\prime }}\sum_{y^{\prime }}\sum_{z^{\prime }}A\left(x^{\prime },y^{\prime },z^{\prime }\right)\; & & \;\\ & \cdot \text{DPK}\left(x-x^{\prime },y-y^{\prime },z-z^{\prime }\right) \end{array}$$

where λ is the decay constant of ^90^Y, *A* represents the 3-D activity concentration voxel space and DPK is the computed DPK per disintegration of ^90^Y, that was computed as previously described. The process described is similar to the DPK convolution scheme used for dosimetry in several recent ^90^Y PET publications (6–11).

### Transformation of images using the local deposition method

Using the LDM, no post-processing is required to perform image-based dosimetry following ^90^Y PET/CT. As with DPK convolution, calculation of a committed absorbed-dose is based on the premise that radioembolization is a permanent implant with a relative distribution that does not change following infusion. The other assumption of the LDM is that energy release by decay of ^90^Y within a voxel is deposited locally, within that same voxel. Given a voxel *V* that is determined using ^90^Y PET/CT to have activity concentration *A_0_* following ^90^Y infusion, the derivation for dosimetry performed using the LDM is given in Eqs 2–5.

(2) $$\begin{array}{llll} E_{\text{avg}} & =\overset{\infty }{\underset{0}{\int }}E\times \psi \left(E\right)\text{dE}=0.935\text{ MeV}\; & & \;\\ & =1.498\times 10^{-13}\text{J} \end{array}$$

(3) $$\begin{array}{llll} E_{\text{com}}\left(\frac{\text{J}}{\text{mL}}\right) & =A_{0}E_{\text{avg}}\overset{\infty }{\underset{0}{\int }}e^{-\lambda t}\text{dt}=\frac{A_{0}}{\lambda }\times \left(1.498\times 10^{-13}\right)\; & & \;\\ & =A_{0}\left(\frac{\text{Bq}}{\text{mL}}\right)\times 4.986\times 10^{-8}\left(\text{J}\cdot \text{s}\right) \end{array}$$

(4) $$\begin{array}{ll} D_{90_{Y}}\left(\text{Gy}\right) & =\frac{A_{0}\left(\frac{\text{Bq}}{\text{mL}}\;\right)\times 4.986\times 10^{-8}\left(\text{J}\cdot \text{s}\right)}{\rho_{\text{liver}}\left(\frac{\text{kg}}{\text{mL}}\;\right)} \end{array}$$

(5) $$\begin{array}{ll} D_{90_{Y}}\left(\text{Gy}\right) & =A_{0}\left(\frac{\text{Bq}}{\text{mL}}\;\right)\times K_{90_{Y}}\left(\text{Gy}\cdot \frac{\text{mL}}{\text{Bq}}\;\right) \end{array}$$

where, *E*_avg_ is the average energy released per decay of ^90^Y based on the probability density function ψ (*E*) for emission (27) and λ is the Y^90^ decay constant based on a half-life of 64.24 h. *A*_0_ is the initial activity concentration in voxel *V* and *E*_com_ is the committed total energy released per unit volume. If *E* has units Joules (J) and *A*_0_ is in Bq/mL, then *E*_com_ will have units J/mL. The committed absorbed-dose, *D* (Gy) within a voxel can then be obtained by dividing *E*_com_ by the density (ρ) of liver tissue in kg/mL, which is defined by the ICRU (22). In Eq. 5, the constants in Eq. 4 have been grouped into a conversion factor, *K* for ^90^Y.

Using these relationships, we have compiled a set of *K* factors for use with the LDM that allow for direct conversion of activity concentration to committed absorbed-dose using Eq. 6 below. Since acquisition workstation software packages for many PET/CT systems do not yet support ^90^Y as a viable PET radionuclide, it may be prudent to perform the scan using one of the other positron emitters listed in Table 3. ^22^Na, ^86^Y, and ^68^Ge have all been used in published literature, although ^22^Na is the most straightforward choice. If the PET scanner directly supports ^90^Y as a viable PET radionuclide, the exponential term in Eq. 6 drops out and the committed absorbed-dose (Gy) using the LDM becomes the product of the activity concentration and conversion factor, *K*. If another radionuclide from Table 3 is used and the delay between infusion and imaging exceeds a few hours, the exponential term becomes important.

(6) $$D_{90_{\text{Y}}}\left(\text{Gy}\right)=A_{0}\left(\frac{\text{Bq}}{\text{mL}}\;\right)\times K_{\text{X}}\left(\text{Gy}\cdot \frac{\text{mL}}{\text{Bq}}\;\right)\cdot e^{\lambda_{c}\cdot t}$$

where, *t* is the time in hours from ^90^Y infusion to the PET scan, *K*_X_ and λ*_c_* are obtained from Table 3. *K* has been computed from the branching ratio of ^90^Y and the radionuclide selected for imaging, X, following Eq. 7 below. The adjusted decay constant, λ*_c_*, is equal to the decay constant of ^90^Y less the decay constant of the radionuclide selected for imaging as described in Eq. 8.

(7) $$K_{\text{X}}\left(\text{Gy}\cdot \frac{\text{mL}}{\text{Bq}}\;\right)=K_{90_{\text{Y}}}\left(\text{Gy}\cdot \frac{\text{mL}}{\text{Bq}}\;\right)\times \frac{\beta_{\text{X}}}{\beta_{90_{\text{Y}}}}$$

where, β_X_ and β_90Y_ are the branching fractions for positron emission for the radionuclide selected for imaging, X, and ^90^Y, respectively.

(8) $$\lambda_{c}=\lambda_{90_{\text{Y}}}-\lambda_{\text{X}}$$

where, λ*_c_* is the corrected decay constant, $\lambda_{90_{\text{Y}}}$ and λ_X_ are the decay constants for ^90^Y and radionuclide X in Table 3.

**Table 3:** **^90^Y dosimetry using PET/CT and the local deposition method**.

Isotope used for imaging	Branching ratio for positron emission	Decay constant λ (h^−1^)	Adjusted decay constant λ*_c_* (h^−1^)	Conversion factor *K*_X_ (Gy-mL/Bq)
^90^Y	0.000032	1.083 × 10^−2^	0	4.782 × 10^−5^
^22^Na	0.905	3.038 × 10^−5^	1.080 × 10^−2^	1.353
^86^Y	0.319	4.702 × 10^−2^	−3.619 × 10^−2^	0.4767
^68^Ge	0.890^a^	1.066 × 10^−4^	1.072 × 10^−2^	1.330

*^a^Branching ratio of daughter, ^68^Ga, which is in secular equilibrium with parent ^68^Ge*.

### Presentation of results

Analysis of the purely mathematical phantom comparison was straightforward since the absence of image noise obviated the need for any statistical analysis. In contrast, analysis of the experimental data for the LDM and DPK at the different reconstruction parameters used required examination for statistical variation. These experimental data were compared to the TDS, which was based off of a mathematical phantom with ideal dosimetry and no noise. Integrated dose-volume histograms (IDVH) were generated for each scanned and mathematical phantom hot-sphere, each dosimetry technique and for all repeated scans. The IDVH is the integral of the DVH from 0 to D, and was selected only because it better illustrated subtle differences in the presented dosimetry techniques. The IDVH is defined as:

(9) $$\text{IDVH}\left(D\right)=\overset{D}{\underset{0}{\int }}\text{DVH}\left(D\right)\text{dD}$$

where DVH is the differential DVH and *D* is the dose in Gy.

A spherical volume of interest (VOI) centered on, but 20% larger than each hot-sphere was used to generate each IDVH. A VOI which slightly exceeded the size of the hot-sphere was selected to ensure that sphere edges were fully included in the IDVH since voxels near the edges are likely to be affected most by the different dosimetry techniques considered. An analysis of the error introduced by the effects of quantum mottle was performed using a one sample Student’s *t*-test, independently comparing the IDVH for LDM and DPK to the TDS, respectively. A two sample Student’s *t*-test was used to determine if statistical differences existed between the IDVH of the LDM and DPK.

## Results

### Results from physical phantom

**Figure 3:**
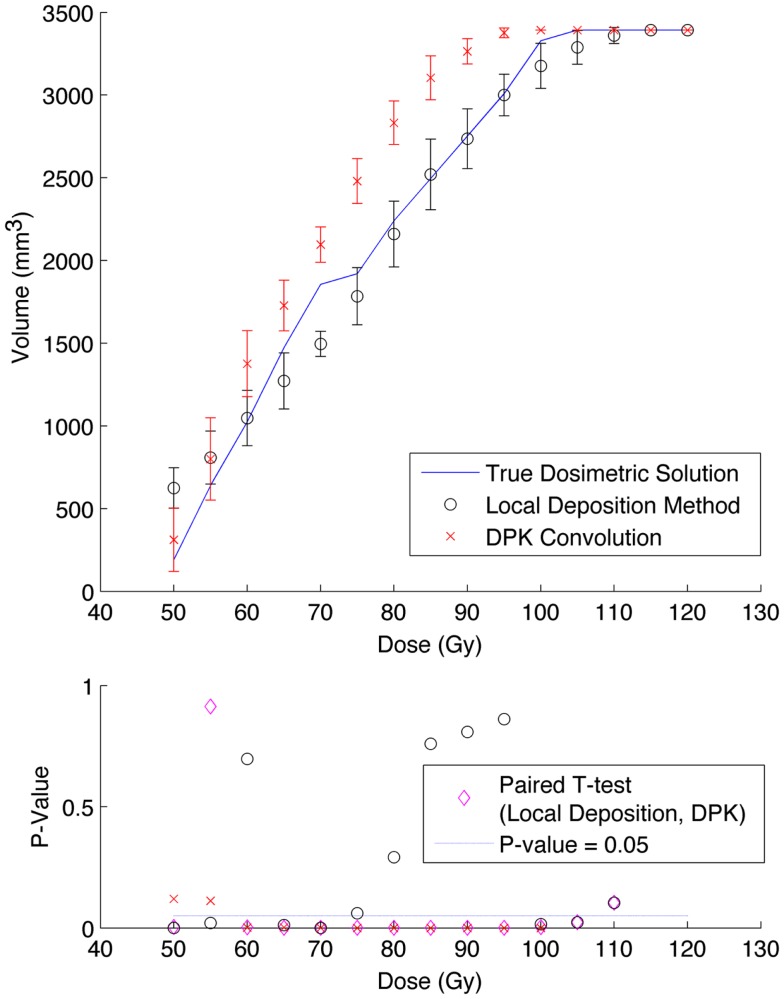
**Top: IDVH of NEMA phantom 17 mm hot-sphere comparing DPK convolution and the local deposition method, as determined from 8, 20 min scans according to recon 1 in Table 1**. Reconstruction was performed using 4 mm isotropic voxels with 1 iteration, 21 subsets, and TOF. Both experimental IDVH are compared to the true dosimetric solution IDVH. Bottom: error analysis.

**Figure 4:**
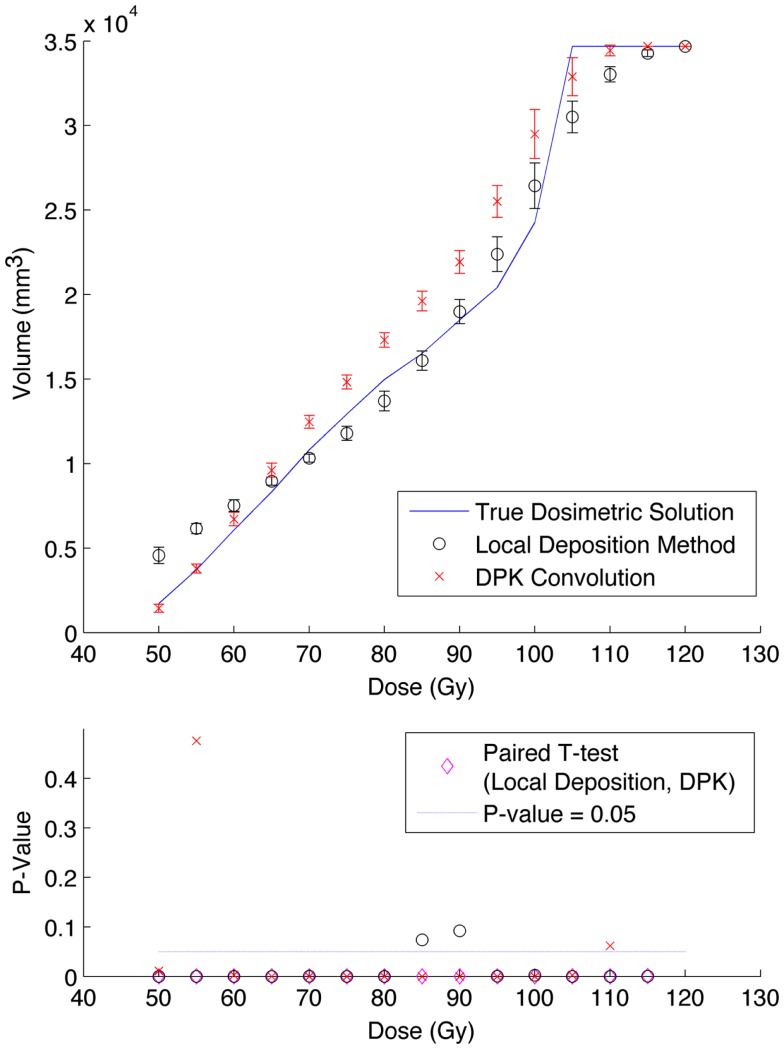
**Top: IDVH of NEMA phantom 37 mm hot-sphere comparing DPK convolution and the local deposition method, as determined from 8, 20 min scans according to recon 1 in Table 1**. Reconstruction was performed using 4 mm isotropic voxels with 1 iteration, 21 subsets, and TOF. Both experimental IDVH are compared to the true dosimetric solution IDVH. Bottom: error analysis.

A comparison of the IDVH for the LDM and DPK using data from reconstruction 1 (Table 1) with 4 mm^3^ isotropic voxels is presented in Figures 3 and 4 for the 17 and 37 mm hot-spheres, respectively. The IDVH for the LDM appeared to more closely follow the shape of the TDS for both hot-spheres, although agreement for the 17 mm sphere was better than the 37 mm sphere. Out of 15 energy bins in the IDVH for the 17 mm sphere, eight LDM bins showed statistically significant differences with the TDS (*p* < 0.05), while 13 DPK bins showed statistically significant differences with the TDS. Statistically significant differences (*p* < 0.05) between LDM and DPK were found in 13 out of 15 energy bins. Results were less clear for the 37 mm hot-sphere, where LDM and DPK both showed statistically significant differences with the TDS in 13 out of 15 bins and differences between LDM and DPK were found in every energy bin. While graphical results for other hot-spheres have not been included, they followed a similar trend where differences between LDM and DPK were detectable for smaller hot-spheres, with LDM providing a slight edge in accuracy in some cases.

**Figure 5:**
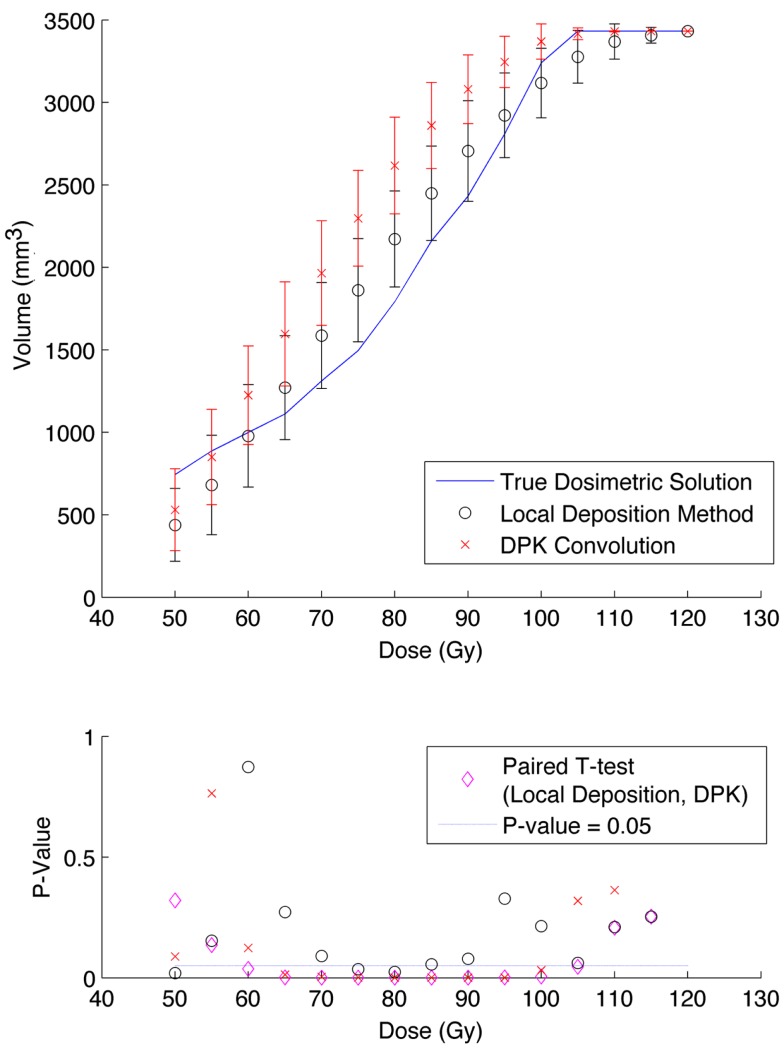
**Top: IDVH of NEMA phantom 17 mm hot-sphere comparing DPK convolution and the local deposition method, as determined from 6, 40 min scans according to recon 2 in Table 1**. Reconstruction was performed using 2 mm isotropic voxels with 1 iteration, 21 subsets, and TOF. Both experimental IDVH are compared to the true dosimetric solution IDVH. Bottom: error analysis.

**Figure 6:**
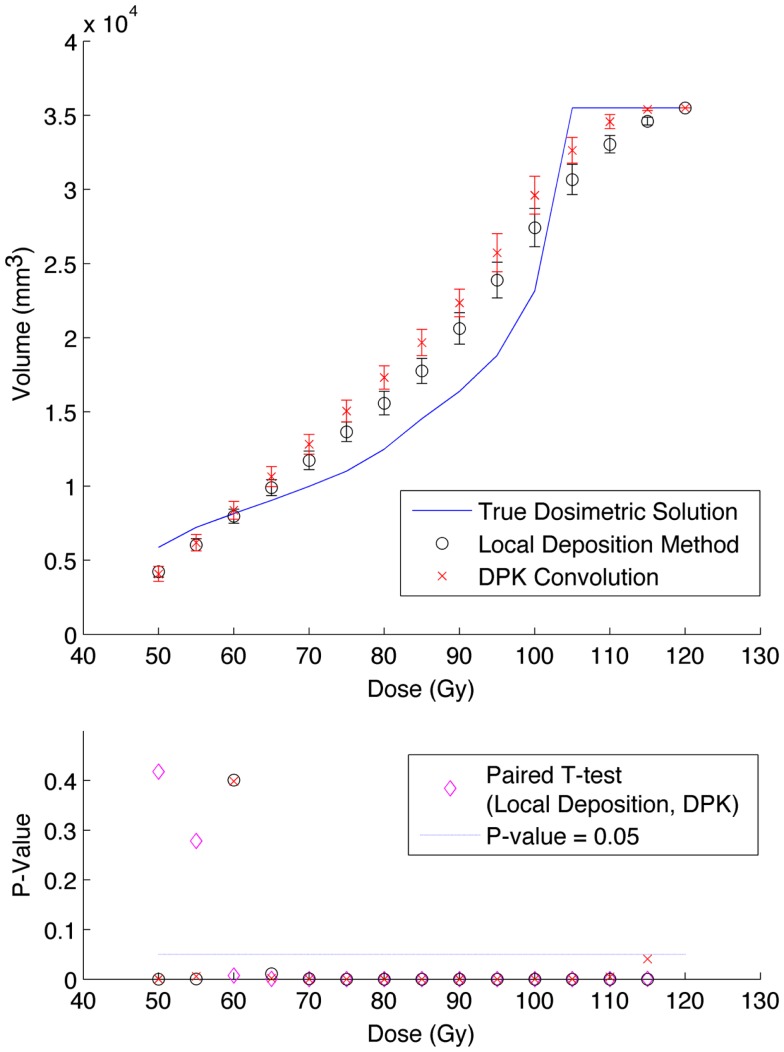
**Top: IDVH of NEMA phantom 37 mm hot-sphere comparing DPK convolution and the local deposition method, as determined from 6, 40 min scans according to recon 2 in Table 1**. Reconstruction was performed using 2 mm isotropic voxels with 1 iteration, 21 subsets, and TOF. Both experimental IDVH are compared to the true dosimetric solution IDVH. Bottom: error analysis.

The phantom results from reconstruction 2 (Table 1) contained higher image noise owing to the 2 mm^3^ isotropic voxel size compared with reconstruction 1, despite the longer scan time. This additional image noise resulted in wider error-bars on the IDVH data presented in Figures 5 and 6 for both the 17 and 37 mm hot-spheres, respectively. Although LDM appeared to more closely match the IDVH of the TDS, it is important to note that both LDM and DPK tended to underestimate the dose compared to the TDS. Out of 15 energy bins in the IDVH for the 17 mm sphere, only 4 LDM bins showed statistically significant differences with the TDS (*p* < 0.05), while 10 DPK bins showed statistically significant differences with the TDS. For the 37 mm hot-sphere, all but one energy bin was statistically different from the TDS for both LDM and DPK. Statistically significant differences between LDM and DPK were found in 11 and 13 energy bins for the 17 and 37 mm hot-spheres, respectively. Once again, in other hot-spheres for which figures are not presented, the increased accuracy afforded by LDM was exaggerated for smaller hot-spheres using 2 mm voxels compared to 4 mm voxels. However, these differences quickly diminished as hot-sphere size increased owing to the effects of image noise.

**Figure 7:**
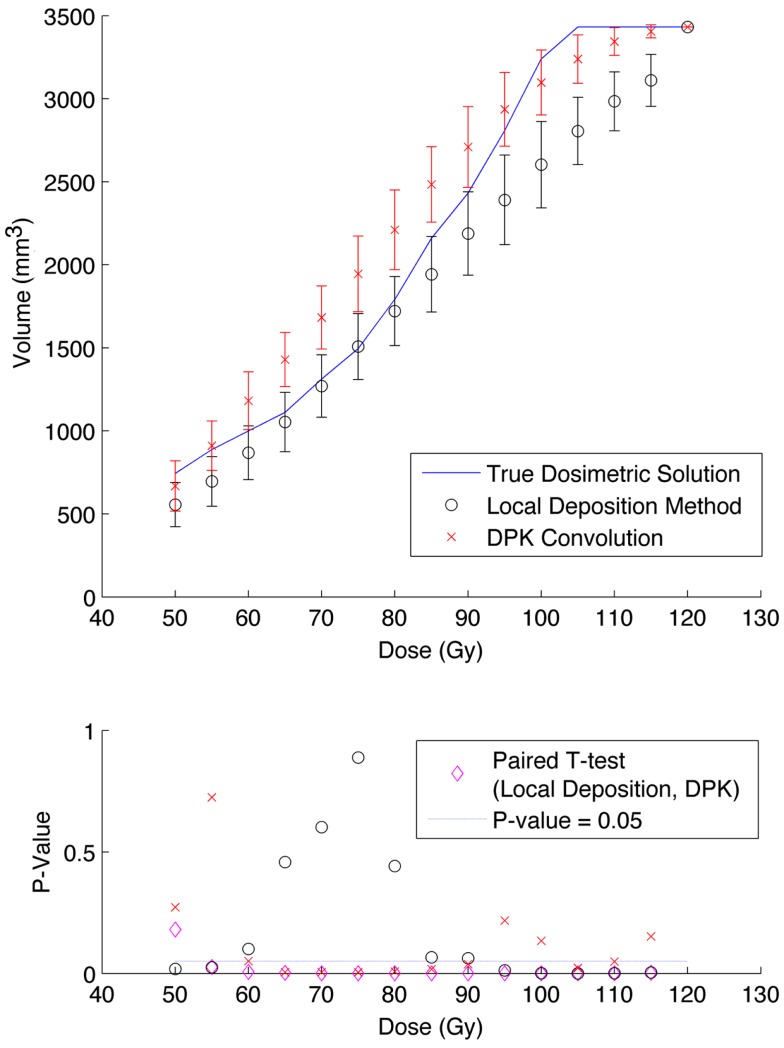
**Top: IDVH of NEMA phantom 17 mm hot-sphere comparing DPK convolution and the local deposition method, as determined from 6, 40 min scans according to recon 3 in Table 1**. Reconstruction was performed using 2 mm isotropic voxels with 1 iteration, 21 subsets, and TOF + RR. Both experimental IDVH are compared to the true dosimetric solution IDVH. Bottom: error analysis.

**Figure 8:**
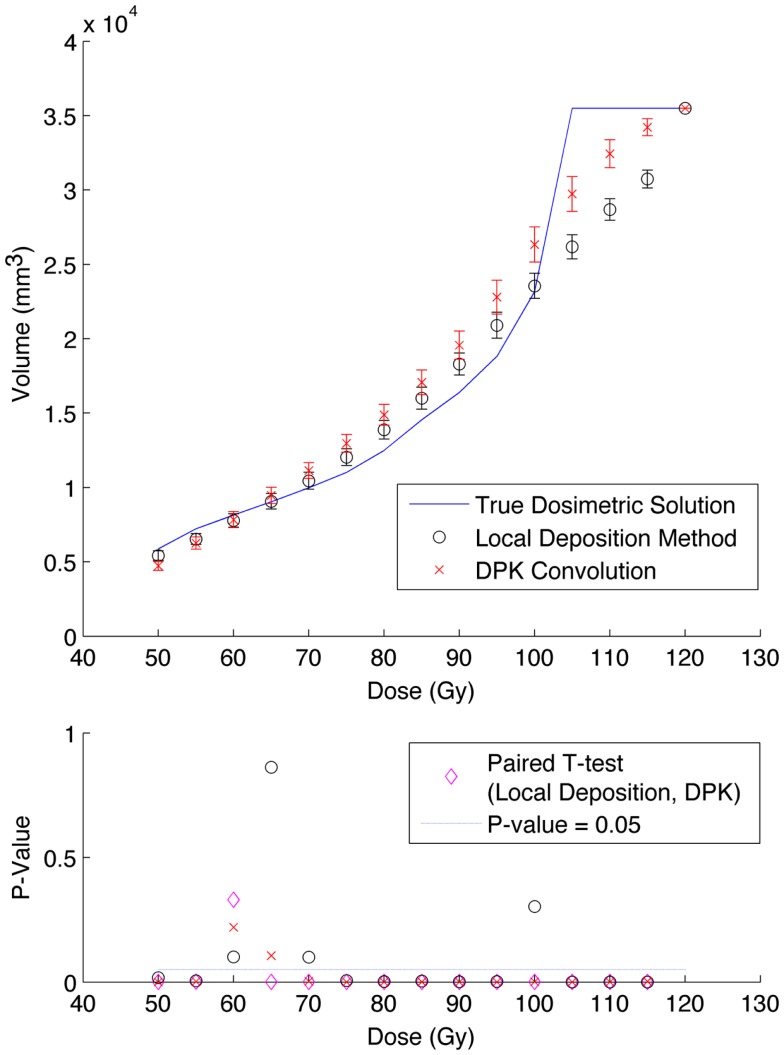
**Top: IDVH of NEMA phantom 37 mm hot-sphere comparing DPK convolution and the local deposition method, as determined from 6, 40 min scans according to recon 3 in Table 1**. Reconstruction was performed using 2 mm isotropic voxels with 1 iteration, 21 subsets, and TOF + RR. Both experimental IDVH are compared to the true dosimetric solution IDVH. Bottom: error analysis.

Results from reconstruction 3 (Table 1) produced images with the greatest amount of noise, due to the use of both a small 2 mm voxel size and RR. Unlike the previous two examples (reconstructions 1 and 2), there does not appear to be any substantial visual accuracy advantage in either LDM or DPK when compared to the TDS. Figures 7 and 8 show the IDVH for the 17 and 37 mm hot-spheres, respectively. These figures indicate that the LDM out performs DPK for doses below 80 Gy, while DPK tended to outperform LDM for doses above 80 Gy. Eight LDM bins showed statistically significant differences with the TDS (*p* < 0.05) while nine DPK bins showed statistically significant differences with the TDS for the 17 mm hot-sphere. Following the same trend, differences between LDM, DPK and the TDS were statistically significant for 11 and 13 bins for the 37 mm hot-sphere, respectively. While these data would indicate that LDM might have slightly outperformed DPK, a visual analysis of Figures 7 and 8 suggests that similar inaccuracies are associated with both methods.

### Results from mathematical phantom

The use of mathematical phantoms allowed for an exact comparison of LDM and DPK, eliminating the effects of image noise. This process also allowed a mechanism to simulate the effect of varying scanner PSF FWHM and reconstruction voxel size.

**Figure 9:**
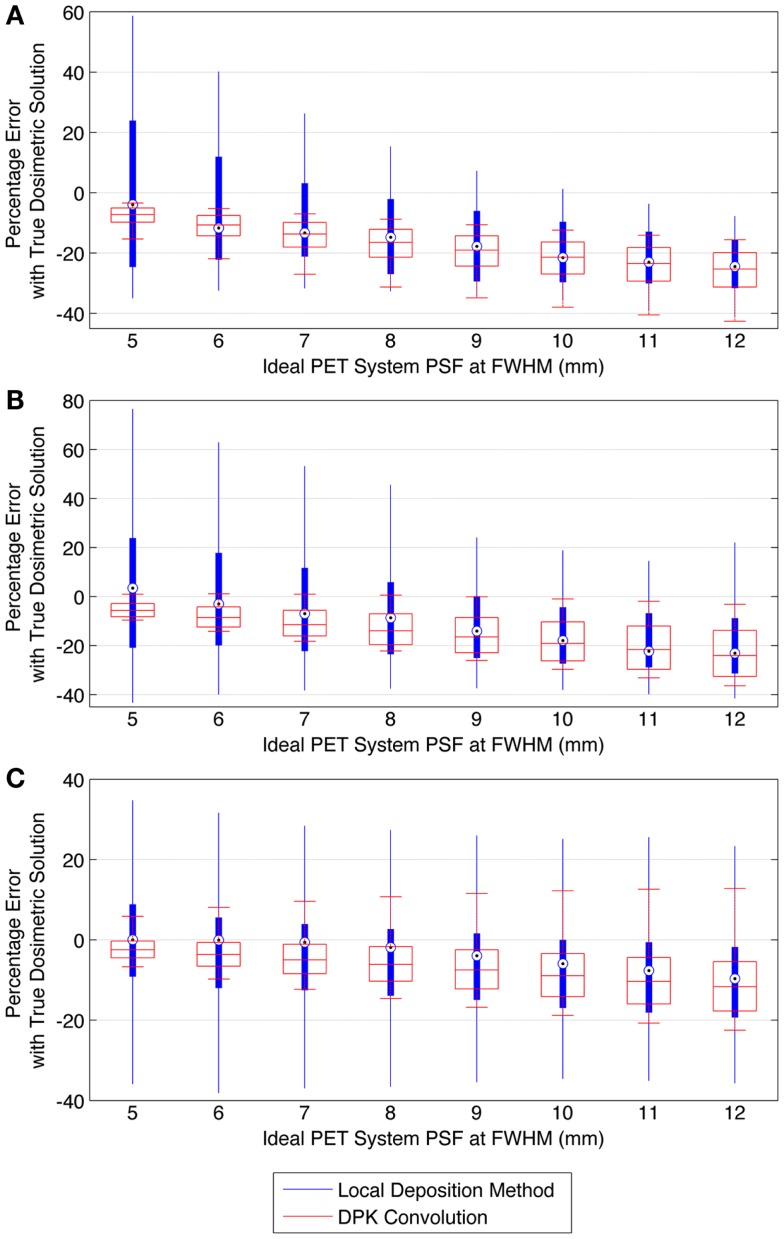
**Comparison of error using LDM or DPK convolution with the TDS based on mathematical phantoms with no noise and ideal PET scanner PSF for a 4 mm isotropic voxel size**. For each simulated PSF, percentage errors between either the LDM or DPK DVH and the TDS DVH for every dose (*D*) in the DVH were computed. Boxplots indicate mean error, 25th and 75th percentile of the distribution as well as upper and lower extremes across all doses, *D*. **(A)** 10 mm hot-sphere, **(B)** 17 mm hot-sphere, and **(C)** 37 mm hot-sphere.

Figure 9 describes the results of the mathematical phantom comparison for an idealized shift-invariant scanner PSF ranging from 5.0 mm at FWHM to 12.0 mm when reconstructed with 4 mm^3^ isotropic voxels. The simulation was performed at 0.25 mm PSF FWHM increments within this range; however, data were plotted at 1 mm increments for ease of interpretation. In Figure 9, the relative performance of an entire DVH obtained for a specific idealized scanner PSF has been compressed into a single boxplot. This has been done for the LDM using Eq. 10 below:

(10) $$\forall D:\text{Erro}{\text{r}}_{\text{LDM}}\left(D\right)=\frac{\text{DV}{\text{H}}_{\text{LDM}}\left(D\right)-\text{DV}{\text{H}}_{\text{TDS}}\left(D\right)}{\text{DV}{\text{H}}_{\text{TDS}}\left(D\right)}\cdot 100$$

where Eq. 10 was executed for all absorbed-doses (*D*) in the DVH’s corresponding to the TDS and LDM. A similar relationship was used to find the error associated with the DPK dosimetry technique. The box plots in Figure 9 describe the mean error, upper and lower quartile, and extreme limits from Eq. 10 for each simulated scanner PSF and for both LDM and DPK. For all hot-spheres, the average magnitude of the DVH error was less when LDM was used, possibly confirming experimental findings. In addition, the accuracy of LDM improved with decreasing scanner PSF FWHM. The mean error decreased as hot-sphere size increased, a finding that is contrary to the experimental comparison. Ideal scanner PSF FWHM <5.5 mm at FWHM resulted in a mean error of <10% for all hot-sphere sizes, when the LDM was used. However, the upper and lower extremes in the error for the LDM were large, exceeding the upper and lower extremes for DPK in most cases.

**Figure 10:**
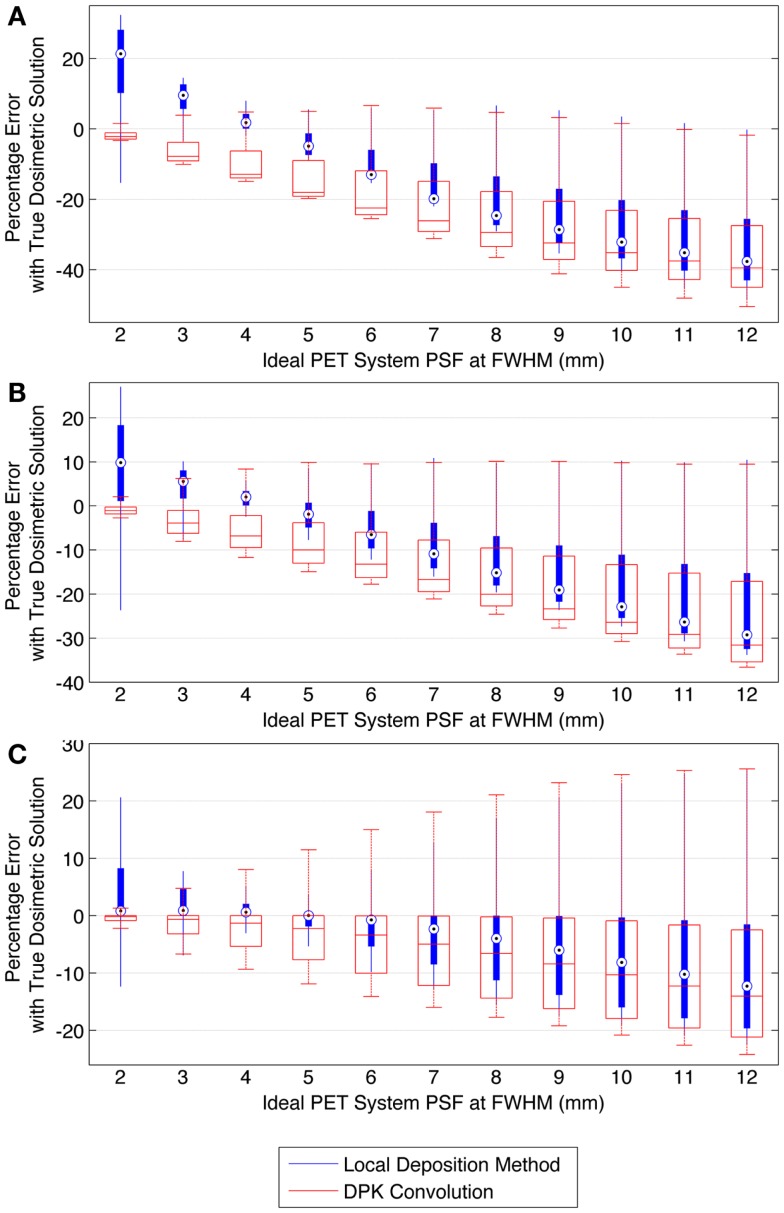
**Comparison of error using LDM or DPK convolution with the TDS based on mathematical phantoms with no noise and ideal PET scanner PSF for a 2 mm isotropic voxel size**. For each simulated PSF, percentage errors between either the LDM or DPK DVH and the TDS DVH for every dose (*D*) in the DVH were computed. Boxplots indicate mean error, 25th and 75th percentile of the distribution as well as upper and lower extremes across all doses, *D*. **(A)** 10 mm hot-sphere, **(B)** 17 mm hot-sphere, and **(C)** 37 mm hot-sphere.

When voxel size is reduced to 2 mm^3^, the magnitude of the mean error of the LDM is no longer less than the error of DKP in all scenarios. Figure 10 illustrates the error of LDM and DPK compared to the TDS for 2 mm^3^ isotropic voxels for ideal scanner PSF FWHM ranging from 2.0 to 12.0 mm at FWHM. Analyses of these data show a distinct crossover point where DPK begins to become more accurate than LDM. As intuitively expected, this happens as the scanner PSF FWHM becomes very small. At a PSF FWHM of 3.25 mm, the mean error in DPK and LDM is approximately equal and opposite. Decreasing the PSF FWHM below 3.25 mm results in an increase in the accuracy of DPK and a decrease in the accuracy of LDM. However, for PSFs exceeding 3.25 mm at FWHM, the LDM produced the most accurate results independent of hot-sphere size; although the differences were most obvious for smaller hot-spheres. At a 2 mm^3^ voxel size, a PSF FWHM of 4.5 mm resulted in the lowest mean error in the LDM, which was <3% for all hot-sphere sizes.

## Discussion

Several conclusions can be drawn stemming from analysis comparing the physical phantom scans with the mathematical TDS. Experimental data from reconstruction 1 and 2 indicated possible superior visual agreement between the TDS and the LDM, which was also confirmed by the included statistical analysis. However, even accounting for the variation from scan to scan due to quantum mottle, statistically significant differences between both dosimetry methods and the TDS were evident in many IDVH energy bins. These differences can be visually noted by comparing the phantom scan results to the TDS noting error-bars in Figures 3–8. This highlights an important finding that the magnitude of the quantum noise rather than statistical variations from scan to scan has a significant effect on the shape of the DVH. Even in areas of homogeneous ^90^Y uptake, increasing image noise serves to broaden the shape of the DVH, resulting in segments of the volume where the dose is underestimated, and segments where it is overestimated. This is evident on analysis of the IDVH of both the LDM and DPK. Note that when 4 mm^3^ voxels are used (Figures 3 and 4) the shape of the IDVH of experimental methods more closely matches the TDS than when smaller voxels are used (Figures 5–8) since the magnitude of the noise is degreased, owing to the larger voxel size. However, even with 4 mm^3^ voxels, over-estimation of the IDVH at low doses (~50–60 Gy) and underestimation of the IDVH at high doses ( >100 Gy) can be seen in Figures 3 and 4. Decreasing the voxel size to 2 mm^3^ increased image noise, exacerbating these effects. Figures 5 and 6 show a flattening of the slope of the IDVH due to the higher noise magnitude associated with 2 mm^3^ voxels. Finally, the largest component of image noise, with 2 mm^3^ voxels and RR resulted in the greatest flattening of the IDVH slope as illustrated in Figures 7 and 8. The magnitude of the image noise in a ^90^Y PET/CT scan contributes to a larger error with the TDS than differences between DPK and LDM dosimetry techniques in most circumstances. While LDM appears to offer a slight improvement in accuracy compared to DPK on both visual and statistical comparisons, the dominant contributor to error in both cases is the flattening of the IDVH by image noise.

The mathematical phantom comparison described the performance of the dosimetry techniques in the absence of image noise under ideal conditions. Several findings mirrored the results of the physical phantom analysis. Figures 9 and 10 compares the error in DVH between the LDM and TDS and DPK and TDS, both as a function of idealized scanner PSF. When 4 mm^3^ voxels were used (Figure 9) LDM outperformed DPK, although the differences were small. Overall errors for both dosimetry techniques were greater as hot-sphere size decreased and also as the ideal scanner resolution worsened (increasing PSF FWHM). Additionally, the differences in the LDM and DPK were reduced as the scanner resolution worsened. When the voxel size was decreased to 2 mm^3^, the differences between DPK and LDM became more obvious. LDM substantially outperformed DPK for idealized scanner PSFs >3.25 mm at FWHM, regardless of hot-sphere size. Just as with 4 mm^3^ voxels, the accuracy differences between LDM and DPK were reduced as scanner resolution decreased (larger PSF FWHM). At a PSF of 3.25 mm at FWHM, the error introduced by DPK and LDM is approximately equal and opposite, and as the PSF decreases, DPK will provide the most accurate solution. However, for a PSF that is >3.25 mm at FWHM, the blur introduced by the scanner PSF combined with DPK convolution will result in over-estimation of the distribution of β-energy deposition away from the site of decay. In these cases, the scanner PSF alone and thus the LDM provides a better approximation of β-dose deposition. It should be noted that while image noise had a profound effect on the phantom scans, there are some findings from the 2 mm^3^ mathematical comparison that are mirrored in reconstructions 2 and 3 of the physical scan. As illustrated in Figure 2, the PET/CT scanner used in this work has a PSF for ^90^Y using RR of 3.1 mm at FWHM. While not shown in Figure 2, the FWHM without RR using the same measurement and reconstruction parameters as the data in Figure 2 was 5.5 mm at FWHM. The physical results of reconstruction 3 show equivalent accuracy for both LDM and DPK, mirroring the finding from the mathematical phantom, which indicated errors will be approximately equal and opposite at a PSF FWHM of 3.25 mm for LDM and DPK, respectively. On the other hand, without RR, the LDM demonstrated a potential advantage following physical results from reconstruction 2 as shown in Figures 5 and 6. Once again, this mirrors mathematical phantom data in Figure 10, suggesting that LDM will be more accurate at a FWHM of 5.5 mm. Given the expected ^90^Y resolution of modern PET/CT systems (Table 2), there is mathematical evidence that suggests LDM will outperform DPK. However, it is likely that the high image noise associated with ^90^Y PET/CT may obscure much of the difference between the two methods.

## Conclusion

The LDM is an alternative image-based dosimetry technique for ^90^Y radioembolization. It has the benefit of simplistic implementation, requiring no post-processing. The conversion factors in Table 3 can be used to transform raw ^90^Y PET/CT pixel values representative of activity concentration into committed absorbed-dose. Mathematical evidence suggests that in certain cases, the LDM will out-perform conventional DPK convolution. However, ^90^Y PET/CT image noise will likely obscure much of the realized theoretical benefit. Nevertheless, due to its ease of use and potential for improved accuracy, the LDM is a viable option for routine post-radioembolization ^90^Y dosimetry based on PET/CT imaging.

## Conflict of Interest Statement

Alexander S. Pasciak has an actively funded educational grant from SIRTeX Medical, USA, however, none of the work presented in this manuscript is directly related to this grant. Austin C. Bourgeois and Yong C. Bradley declare no conflict of interest.
